# The research beamlines at the Dresden proton therapy facility: available infrastructure and experimental capabilities

**DOI:** 10.3389/fonc.2025.1594973

**Published:** 2025-06-05

**Authors:** Felix Horst, Elisabeth Bodenstein, Michael Baumann, Elke Beyreuther, Jozef Bokor, Wolfgang Enghardt, Sebastian Gantz, Martin Hejzlar, Fritz Kurth, Markus Meyer, Stefan Pieck, Christian Richter, Jörg Pawelke

**Affiliations:** ^1^ Institute of Radiooncology - OncoRay, Helmholtz-Zentrum Dresden-Rossendorf, Dresden, Germany; ^2^ OncoRay - National Center for Radiation Research in Oncology, Faculty of Medicine and University Hospital Carl Gustav Carus, Technische Universität Dresden and Helmholtz-Zentrum Dresden-Rossendorf, Dresden, Germany; ^3^ Deutsches Krebsforschungszentrum, DKFZ, Heidelberg, Germany; ^4^ Institute of Radiation Physics, Helmholtz-Zentrum Dresden-Rossendorf, Dresden, Germany; ^5^ Ion Beam Applications (IBA), Louvain-la-Neuve, Belgium; ^6^ Department of Radiotherapy and Radiation Oncology, Faculty of Medicine and University Hospital Carl Gustav Carus, Technische Universität Dresden, Dresden, Germany; ^7^ Central Department of Research Technology, Helmholtz-Zentrum Dresden-Rossendorf, Dresden, Germany

**Keywords:** proton therapy, experimental room, beamline, translational research, proton beam

## Abstract

The proton therapy facility in Dresden, Germany, has one treatment room equipped with a rotating gantry where patients are treated and an experimental room equipped with two horizontal beamlines for translational research. The present work describes the technical characteristics and provides measured beam data of these two complementary beamlines, one delivering scanned beams with quasi-clinical parameters and the other one stationary continuous and pulsed pencil beams with parameters exceeding the clinically used range. Features of the facility are the large scale of the experimental room enabling the development and installation of large devices and the parallel beam operation with the clinical room allowing irradiation experiments on weekdays and during daytime. An overview of past and ongoing physics and biology experiments performed at the facility by internal and external researchers from academia and industry is given, demonstrating its versatile experimental capabilities. This includes the development of novel proton therapy approaches and technology as well as elaborate *in-vitro* and *in-vivo* small animal experiments for which the necessary infrastructure is available in the same building.

## 1 Introduction

Over the last few decades proton therapy has developed from a niche radiotherapy application, being offered at only a few centers worldwide, to a radiotherapy modality frequently employed to treat several indications. Today there are more than 100 proton therapy centers in operation worldwide and many more are planned or under construction. However, to this day the field of proton therapy is strongly shaped by innovations in all disciplines involved such as accelerator and beamline development, improvements in treatment planning and beam application, dosimetry and detector development, integration of image guidance and verification systems as well as radiobiological research. While there are proton therapy centers that focus solely on patient treatment, other facilities also have their own research programs (1), especially those connected to universities or university hospitals. There are many examples of proton (and also heavy ion) therapy centers that use their beamlines for research (2–12). Some of these centers have dedicated experimental rooms while at others the beamlines located in the treatment rooms are shared between researchers and patient treatment.

Besides the proton accelerator (isochronous cyclotron) and the beam transport system, the Dresden proton therapy facility has two treatment rooms: a room for clinical treatments with a 360° gantry system and an experimental room with two horizontal beamlines, of which one is equipped with a dedicated pencil beam scanning (PBS) nozzle. The clinical part of the proton facility is operated as *University Proton Therapy Dresden* (UPTD) by the Carl Gustav Carus University Hospital Dresden. It has been in operation since 2014 and is one out of five clinical particle therapy centers in Germany. The experimental room is operated by OncoRay - National Center for Radiation Research in Oncology, a research platform that is jointly operated by Carl Gustav Carus university hospital, the medical faculty of the Technical University Dresden and the Helmholtz Center Dresden-Rossendorf. The research program at OncoRay has a strong translational focus and covers all levels, from basic research (e.g. *in-vitro* radiobiology experiments) and pre-clinical studies (e.g. radiobiological *in-vivo* tests in small animals) toward actual clinical application in patients (e.g. development of prompt gamma based range verification systems).

The exceptionally large experimental room (length: 19.4 m, width: 14.3 m, height: 5.0 m, about 250 square meter usable area) allows the testing and development of large-scale devices (e.g. in-beam MRI scanner prototypes). Inside this experimental room, two horizontal proton beamlines are available. The first beamline has provided static pencil beams (fixed beam beamline, FB-BL) for experiments since 2014. Dedicated hard- and software for this beamline were developed in-house. A software with a user interface to make a beam request (e.g. specific energy and beam current) is connected to the main control system of the proton therapy system and allows the use of the full beam parameter range. The second beamline is equipped with a PBS nozzle (pencil beam scanning beamline, PBS-BL) and has been in operation since 2019. The irradiation at the PBS-BL is done via the beam control system of the manufacturer.

This article describes the technical characteristics of the two beamlines available in the experimental room of the Dresden proton therapy facility as well as the available infrastructure, referred to together as the experimental area. Furthermore, measured beam data that are relevant for users are provided and examples of past and ongoing experimental activities are presented.

## 2 Overview of experimental area

### 2.1 Overview of the Dresden proton therapy facility

**Figure 1:**
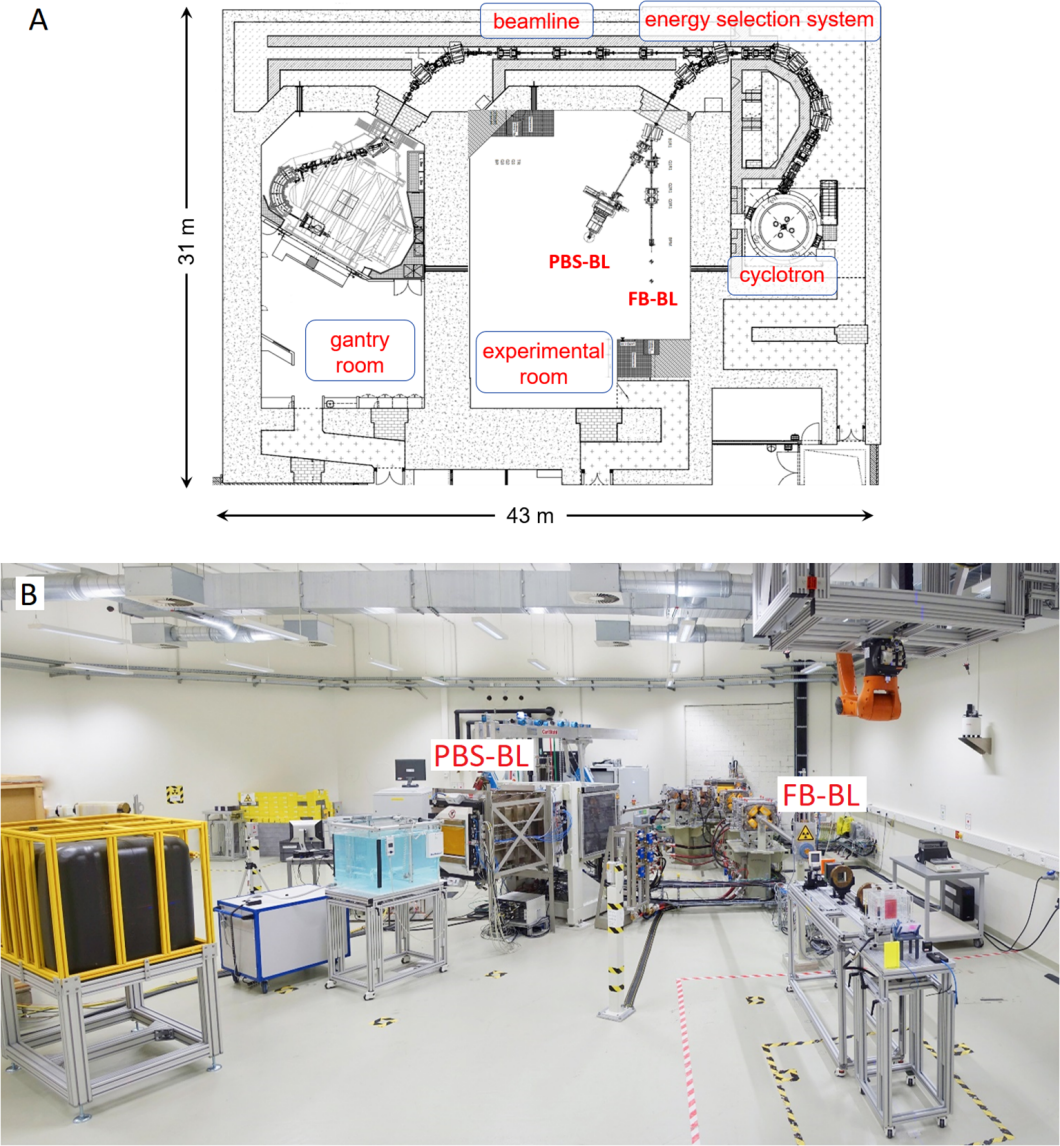
**(A)** Layout of the Dresden proton therapy facility including the cyclotron, the energy selection system, the gantry treatment room and the experimental room with its two beamlines (FB-BL and PBS-BL). **(B)** Photo of the beamline section in the experimental room. A water phantom for 3D dosimetry is set up at the PBS-BL while at the FB-BL a double scattering setup (13) used for irradiation of cell culture flasks can be seen. At the PBS-BL a large water tank (black tank, in yellow cage) used as beam dump is visible as well. At both beamlines quadrupole magnets are used to focus the proton beam. The scanning dipole magnets at the PBS-BL that steer the beam horizontally and vertically are hidden in the white frame upstream of the nozzle.

The Dresden proton therapy facility is equipped with a ProteusPLUS proton therapy system (IBA, Louvain-la-Neuve, Belgium), shown schematically in **Figure 1A**. It is based on a 230 MeV isochronous cyclotron (IBA C230) (14, 15) that provides beam currents up to 500 nA which is the radiation protection limit of the facility. The primary 230 MeV protons are guided through a degrader-based energy selection system (aluminum, carbon or beryllium degraders depending on the energy) with achromatic dipoles and slits that are used to adjust the emittance and energy spread of the degraded proton beams (16). This allows the selection of energies in the range of 70 − 226.7 MeV which are then transported via a beamline to the gantry treatment room or steered directly into the experimental room. Inside the experimental room one of two beamlines (FB-BL or PBS-BL) can be selected for irradiation. **Figure 1B** shows a photo of these two beamlines inside the experimental room with example setups.

### 2.2 Beam operation

**Figure 2:**
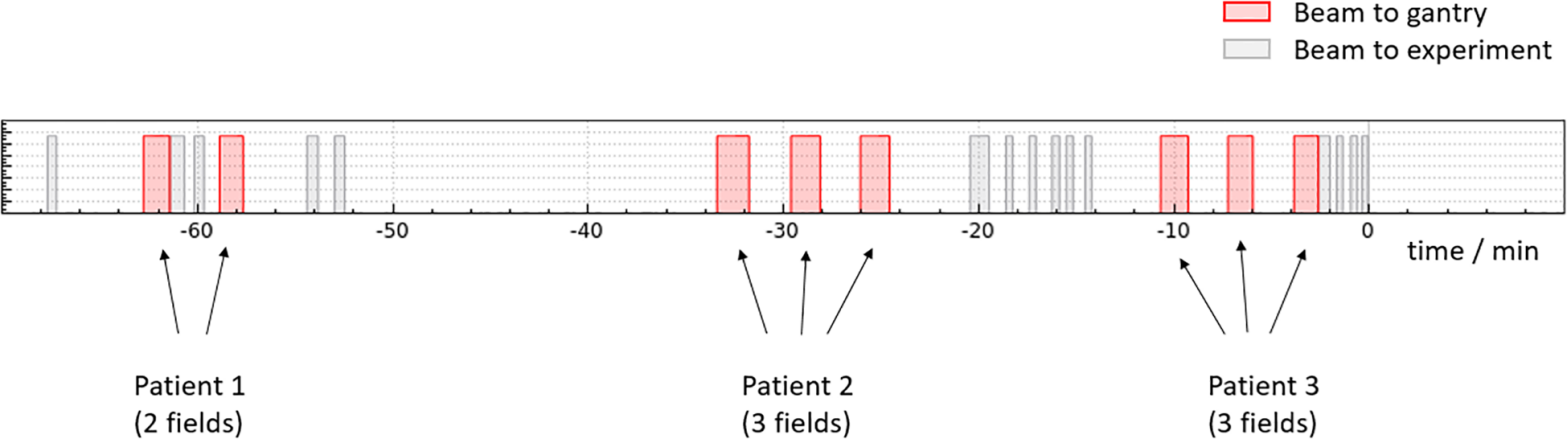
Example diagram showing the sharing of the proton beam the Dresden proton therapy facility between the gantry treatment room and the experimental room. Most patients are treated with multiple fields (in this example the first one with two fields and the next two patients with three fields). In between the fields, gaps of 1 − 2 minutes can be used for an irradiation in the experimental room. The gaps in between the patients are typically 10 min or more (depending how complicated the patient setup is) which can be used for an irradiation in the experimental room as well.

A unique feature of the Dresden proton therapy facility is that experimental activity using the proton beam is possible in parallel with the clinical operation (17, 18). Therefore irradiation experiments are possible from Monday to Friday from 6:30 am until 11:30 pm and on Saturdays from 6:30 am until 9:30 pm. Because only a single gantry room is used for patient treatment and a considerable fraction of the treatment time is used for patient preparation and setup changes between the fields, there is a high beam availability for experiments throughout the day. The irradiation in the experimental room only needs to be interrupted when a patient is ready for treatment and can be continued immediately afterwards. **Figure 2** shows an example of how the experimental room shares the proton beam with the clinical operation in the gantry room.

### 2.3 Research beamlines

The experimental room of the Dresden proton therapy facility has two beamlines as shown in **Figure 1**. **Table 1** compares the relevant technical characteristics of the two beamlines.

**Table 1:** Comparison of relevant parameters of fixed beamline (FB-BL) and beamline equipped with dedicated pencil beam scanning nozzle (PBS-BL).

Parameters	FB-BL	PBS-BL
Proton energy (at laser position)	70–225 MeV	70 – 226.7 MeV
Proton range in water (at laser position)	4.10 – 31.70 g/cm^2^	4.10 – 32.16 g/cm^2^
Time to change proton energy	no automatic energy change	2–6 s
Maximum transmission from cyclotron to beamline exit	~ 42%	~ 13%
Beam current at beamline exit	0.001–210 nA	0.1–5 nA
Beamline height	125 cm	125 cm
Positioning room lasers	1 horizontal, I vertical	2 opposite horizontal, 2 vertical
Vacuum exit to laser position	205 cm	63 cm
Beam spot size at laser position (1*σ*)	8.1 mm at 150 MeV	4.2 mm at 150 MeV
Maximum field size	pencil beam double scattering setup: 10 × 10 cm^2^	pencil beam scanning: 40 cm (horizontal) × 30 cm (vertical)
Dose homogeneity	double scattering setup: ± 2%	pencil beam scanning: ± 1%
Beam pulsing	duration period flexible between 100 *μ*s and minutes	scanned beam (~ ms per spot with ~ ms pauses between spots, ~ s pauses between energy layers)

#### 2.3.1 Characteristics of FB-BL

The FB-BL was the first beamline that was installed in the experimental room of the Dresden proton therapy facility and has been used for experiments since 2014. It provides static pencil beams covering the energy range used for proton therapy at the gantry room (100 − 225 MeV) plus lower energies down to 70 MeV. The beam current can be set to the entire range available from the proton therapy system and is energy-dependent due to the reduction of the transmission through the energy-selection system with decreasing energy. An example transmission curve for the FB-BL is shown in ref (19). Dedicated hardware and software were in-house developed (17, 20), including the beam monitor system employing a segmented transmission ionization chamber (model 34058, PTW, Freiburg, Germany) with appropriate readout electronics, a dedicated current loop module for fast beam pulsing and range setting, a software library to communicate with the IBA system using the available XML-RPC interface and a user-friendly control software (High Energy Beam Control, HEBC). A priority system terminating the irradiation automatically if the gantry room requests the beam has been implemented in order to ensure a smooth parallel operation without disturbing the clinical workflow.

The FB-BL has a high transmission efficiency from cyclotron to beamline exit of about 40% at 225 MeV. This is about a factor 3 higher than for typical beamlines with a PBS nozzle because the divergence slits are completely open, however, at the expense of slightly reduced quality of the beam properties like spot size. This together with the possibility of pulsing the beam with very accurate and reproducible timing, makes the FB-BL especially well-suited for experiments in the ultra-high dose rate (UHDR) regime (21). The dose rate range that has been achieved in previous experimental setups ranged from 10^−6^ Gy*/*s to 600 Gy*/*s. Over the years, different dedicated irradiation setups for different purposes were developed for the FB-BL: for instance a double-scattering setup for homogeneous large fields (Exponat-O) with a transmission efficiency of 30% (13), a setup for irradiation of partial volumes of mouse brains with a sharply collimated proton beam (22) as well as a UHDR spread-out Bragg peak setup using a 3D-printed range modulator (19).

**Figure 3:**
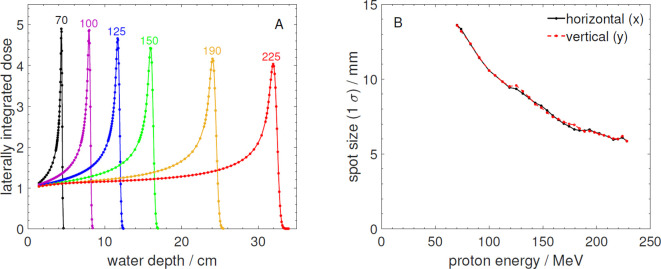
**(A)** Measured laterally integrated depth dose profiles normalized to the entrance measured at the FB-BL. The numbers above the curves are the corresponding proton energies in MeV. **(B)** Measured beam spot size (1*σ*) in horizontal (x) and vertical (y) direction as a function of proton energy at the laser position of the FB-BL.

**Figure 3A** shows Bragg curves for selected energies over the energy range available, measured with a Bragg peak chamber (model 34070-2,5, PTW, Freiburg, Germany) in a water phantom (23) and **Figure 3B** shows the spot size at the room laser position as a function of energy (1*σ* of the Gaussian profiles, in the horizontal and vertical directions) measured using a scintillating screen detector (LynxPT, IBA Dosimetry, Schwarzenbruck, Germany).

#### 2.3.2 Characteristics of PBS-BL

The flexibility of the FB-BL allows a wide range of experiments. However, for certain types of experiments it is beneficial to replicate the clinical beam parameters of scanned proton beams. Therefore, the PBS-BL was installed in the experimental room and is used for experiments since 2019. The PBS-BL is a research beamline that offers the full functionality and beam parameter range used for patient treatment with scanned protons. It is equipped with a dedicated pencil beam scanning nozzle (IBA, Louvain-la-Neuve, Belgium) (24) employing an IC2/3 detector (IBA, Louvain-la-Neuve, Belgium) (25) for monitoring of the beam position and intensity and for controlling the irradiation treatment plans and spot patterns via the PBS technique (16). While the FB-BL is operated using an in-house developed dedicated hardware and software interface connected to the main control system from IBA, the PBS-BL beam delivery relies fully on IBA hardware and software. Irradiation of fields and treatment plans can be performed using different software tools (all by IBA, Louvain-la-Neuve, Belgium): using the adaPT deliver software with DICOM RT files as input, via the BMS standalone software with input files in PLD (PBS Layer Dose) format or using the pristine beam tool for experiments using static pencil beams if needed (e.g. QA or base data measurements). Treatment plans for the PBS-BL can be optimized and dose calculation can be performed using the RayStation treatment planning software (RaySearch Laboratories, Stockholm, Sweden) (26). As shown in **Table 1** the PBS-BL is not as flexible as the FB-BL regarding some parameters (e.g. the beam current range), but it can perform a volume conformal irradiation using the PBS technique in a manner that is comparable to the gantry in the neighboring treatment room. This is for the benefit of research projects that are already in an advanced translational stage, such as the development of an online range verification system using the prompt gamma ray timing method or the development of magnetic resonance imaging (MRI) guided proton therapy. When the beam is requested for patient treatment, priority is given to the gantry. An automatic shutoff of the irradiation like at the FB-BL is not implemented for the PBS-BL.

The PBS-BL has a defined isocenter as reference point (even though the beamline cannot rotate), located 63 cm from the vacuum exit window (24). The isocenter plane is used for calibrating the horizontal and vertical deflection lengths of the scanning magnets and for setting the focusing of the beam optics. A model of the magnetic scanning system of the PBS-BL based on finite element methods combined with Monte Carlo transport simulations was created (27).

Regular machine QA checks, following the clinical practice at the gantry beamline, are performed at the PBS-BL in the experimental room as well. These QA checks control the spot sizes and positions as well as the scanned field homogeneity, proton ranges and absolute dose output, and the stability of these parameters over time.

**Figure 4:**
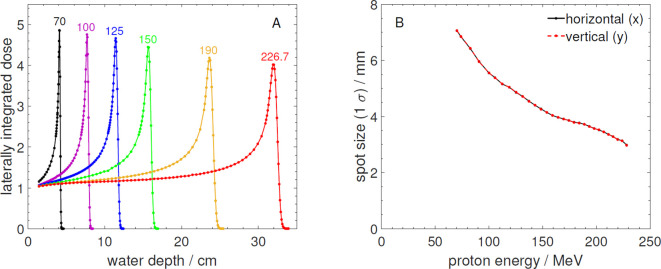
**(A)** Measured laterally integrated depth dose profiles normalized to the entrance measured at the PBS-BL. The numbers above the curves are the corresponding proton energies in MeV. **(B)** Measured beam spot size (1*σ*) in horizontal (x) and vertical (y) direction as a function of proton energy at the beam isocenter position of the PBS-BL. The data was measured with the same detectors like the data in **Figure 3**.

**Figure 4A** shows Bragg curves for selected energies over the energy range available and **Figure 4B** shows the spot size at the isocenter position as a function of energy.

While the Bragg curves are almost identical to those measured at the FB-BL (compare with **Figure 3**), the reported spot sizes at the PBS-BL are smaller and more symmetrical. The smaller spots as well as the better symmetry of the proton beams at the PBS-BL in comparison to the FB-BL are mainly due to its more advanced ion optical design and additionally due to the isocenter point of the PBS-BL being closer to the beam exit window than the reference point defined by the positioning lasers for the FB-BL (see **Table 1**).

**Figure 5:**
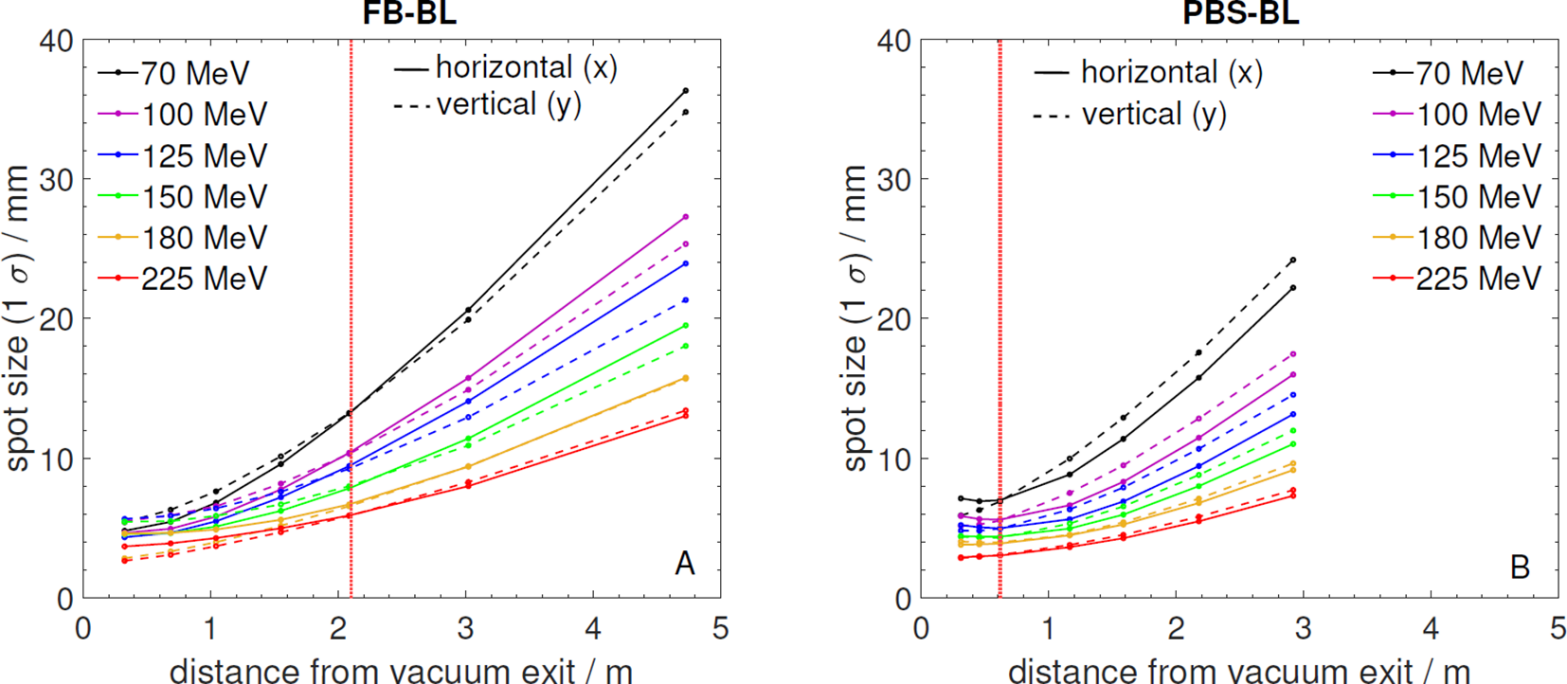
**(A)** Beam spot size (1*σ*) in horizontal (x) and vertical (y) direction measured as a function of distance from the vacuum exit window for selected energies at the FB-BL. The red dotted line marks the reference point indicated by room lasers. **(B)** Beam spot size (1*σ*) in horizontal (x) and vertical (y) direction measured as a function of distance from the vacuum exit window for selected energies at the PBS-BL. The red dotted line marks the beam isocenter indicated by room lasers.

**Figure 5** shows the beam spot size (1*σ*) in horizontal and vertical direction as a function of distance from the vacuum exit window for selected energies between 70 and 225 MeV for both beamlines. Close to the vacuum exit, the convergence of the proton beams due to focusing by the beamline magnets can still be noticed for some energies while with increasing distance the beams diverge due to the angular spread caused by multiple Coulomb scattering in the vacuum exit window, nozzle detectors and air. These scattering effects and thus the widening of the beams are more pronounced for the small proton energies. The spots are perfectly round only at the reference distances used for the ion optical setting while before and after they become slightly elliptic. The PBS beamline has a rather short focus setting while at the FB-BL a smaller convergence angle is set.

## 3 Instrumentation and dosimetry equipment

The experimental area is equipped with a large pool of beam instrumentation devices and dosimetry equipment that can be used to characterize the proton beams.

This comprises standard ionization chambers of essentially all different types (Farmer, Semiflex, Advanced Markus, Roos, PinPoint, micoDiamond, Bragg peak chambers), mostly by PTW Dosimetry (Freiburg, Germany) as well as several precision electrometers for readout (PTW UNIDOS Tango and Keithley model 6514). For 2D dose measurements a LynxPT detector (IBA Dosimetry, Schwarzenbruck, Germany) as well as an Octavius ionization chamber array (model 1500XDR, PTW, Freiburg, Germany) are available. Measurements with these detectors can be performed in air, using various types of plastic phantoms or in water. The proton facility is equipped with commercial water phantoms (model T41051 by PTW and Blue Phantom by IBA Dosimetry) as well as a 3D water phantom developed in-house (suitable for use in magnetic fields). For quick measurements of Bragg peaks with a single irradiation, for example to determine the water equivalent thickness of objects and materials, the multi-layer ionization chambers Giraffe and Zebra (IBA Dosimetry, Schwarzenbruck, Germany) (23) are available.

Both beamlines are equipped with monitor ionization chambers which provide monitor units that are proportional to the irradiated number of protons.

## 4 Experiment infrastructure

The proton irradiation is conducted from a dedicated experiment control room, directly next to the experimental room itself. Separate computers are used to control the FB-BL and PBS-BL. Only one beamline can be operated at a time, which is controlled by the IBA therapy safety system (TSS) software. A TSS switch key must be inserted to activate the beamline that should be operated.

Next to the control room, an experiment preparation room is located which can be used, for example, to mount the setups or prepare samples or animals. Furthermore, a mechanical workshop is located in the same building where experimental equipment, holders, phantoms, setup components, etc., can be produced. All large-scale devices and equipment are on rolls or air-cushion platforms and freely movable in the room to maximize the experimental flexibility.

For radiobiological experiments, laboratories with standard equipment for cell culture (sterile bench, autoclave, centrifuges, microscopes, etc.) and histology are available in house at OncoRay and also within collaborations for external users. Furthermore, the handling and proton irradiation of genetically modified cell cultures is feasible. Approved workflows and setups enable the irradiation of adherent and three-dimensional cell cultures (28), whereby an angulated setup (29) allows for the irradiation of sensitive samples that cannot be positioned upright as necessary at horizontal beamlines. *In-vivo* experiments with proton beams using mice are regularly performed at the OncoRay experimental room. An animal facility on the same floor together with a laminar flow box for clean handling in the preparation room next to the experimental room assure short distances and waiting times for the mice before proton irradiation. Inside the animal facility, an imaging platform consisting of the open-source small animal image-guided radiation therapy (SAIGRT) system for cone-beam CT (30) and a combined nanoScan PET/MRI scanner (1 T, Mediso medical imaging systems, Hungary, Budapest) can be applied for pre-treatment and follow-up imaging of irradiated mice. A dedicated setup (31) allows for positioning of mice at the respective imaging devices and the proton beam while keeping the mice at the same position. In the experimental room, a proton radiography setup based on a CMOS flat panel detector (model C9320DK-02, Hamamatsu Photonics K.K., Hamamatsu City, Japan) can be used for image guidance in small animal experiments (32). It provides projection images that can be used to locate anatomical features (e.g. the skull of a mouse) for aiming with a collimated proton beam exactly at the anatomical area of interest (e.g. the hippocampus in a mouse brain). Another device that can be used for image-guidance of proton irradiation of small animals by means of x-ray computed tomography is the small animal irradiator SmART+ IB (Precision X-Ray Inc., Madison, Connecticut, USA) which is located in the experimental room as well and can be moved on rolls in front of the FB-BL (33). For reference irradiation, several 200 kV x-ray tubes (Maxishot200, Comet Yxlon GmbH, Hamburg, Germany) and dedicated setups for *in-vitro* and *in-vivo* experiments are available in house. Moreover, clinically relevant reference beams, i.e., MV photons and MeV electrons, can be used at the linear accelerators of the radiation therapy department of the Dresden University hospital located in the same building.

## 5 Past and ongoing experimental activities

A large variety of radiobiology experiments have been and are performed at both beamlines (FB-BL and PBS-BL) in the OncoRay experimental room. The existing setups can be applied at both beamlines providing some flexibility to perform *in-vitro* studies in parallel to other (non-biological) experiments. Certain types of biological irradiation can be performed more efficiently at the PBS-BL than at the FB-BL. The larger and flexible field size and the less problematic activation of components, compared to the double scattering system based on lead scatterers and brass collimators used at the FB-BL (13), favor the PBS-BL for *in-vitro* studies with many samples and high doses. Examples of recent *in-vitro* studies dealt with the comparison of radiation response of pancreatic 3D cultures to photon and proton irradiation (34) or the differences in tumor cell plasticity after both treatments (35).

*In-vivo* experiments using mice (22, 36) and zebrafish embryos (37) are only performed at the FB-BL in different settings. The static proton pencil beam is of sufficient size for the partial irradiation of mouse brains (22, 36) or even homogeneous treatment of subcutaneous tumors on mouse ears (38) or legs (31). The extended beam parameters of the FB-BL in combination with the controlled delivery of short pulses allowed for the investigation of ultra-high dose rate effects necessary for FLASH radiotherapy, where dose rates greater than 100 Gy*/*s result in reduced side effects compared to irradiation at conventional dose rates (39–41). Therefore, the FB-BL in the OncoRay experimental room is part of the Dresden platform for ultra-high dose rate radiobiology (21).

The large size of the experimental room enables research activities to be carried out with large-scale devices. This is a crucial prerequisite for the research project on the technical integration of magnetic resonance imaging (MRI) and proton therapy which started in 2015 (42). As initially only the FB-BL was available for this purpose, first experiments with a compact 0.95 T permanent magnet were performed to measure magnetic field-induced effects on proton beams in a tissue equivalent material (43). These studied the electron return effect for proton beams (44) and characterized a high-resolution silicon strip detector under the influence of a magnetic field (45). As a next step, an open, compact 0.22 T MRI scanner was placed in the beam path of the FB-BL to demonstrate a first proof-of-concept for simultaneous proton pencil beam irradiation and MR image acquisition (46). Because the FB-BL enables the use of very high beam currents and dose-rates, the first-ever experiments for proton beam visualization were performed with this in-beam MRI scanner (47). After the PBS-BL became available, experiments with the 0.22 T in-beam MRI scanner were performed to quantify interference effects on the MR magnetic field and image quality during active proton pencil beam scanning (48). After a second open in-beam MRI scanner with a field strength of 0.32 T was installed in the beam path of the PBS-BL in 2020, a first systematic characterization of magnetic field-induced dose distortions was performed (49). In 2023, a 0.5 T whole-body, rotatable in-beam MRI scanner was installed, which is capable of real-time imaging (50). With this third in-beam MRI prototype, initial imaging and irradiation experiments are currently being carried out aiming for the characterization and commissioning of this device.

Another example, highlighting the necessity of diverse and variable beam parameters within translational research, is the development of a treatment verification system for clinical use in proton therapy (51). Prompt Gamma-Ray Timing (PGT) (52) utilizes the temporal distribution of prompt gamma radiation generated through nuclear interactions within the patient to infer the range of therapeutic protons. A clinical-grade detector system was developed (53), which was systematically characterized under a wide variety of beam parameters (54) available at the FB-BL. The granular adjustability of beam currents over several orders of magnitude and the capability to apply flexible beam pulsing at this beamline enabled a detailed understanding of the load and amplification behavior (55), as well as other properties of the PGT detectors (53, 54, 56). The flexibly adjustable beam energies, currents, and pulsing were also indispensable for the development and characterization of new proton bunch monitors (57, 58), which are employed as independent timing references for the PGT method. As part of the clinical translation of the PGT system, the experimental setups were progressively adapted to better reflect clinical conditions. For this purpose, the PBS-BL, with its dedicated scanning nozzle and clinical beam control system, provides an ideal foundation. Furthermore, the large experimental room is also used for experiments to determine proton-induced nuclear reaction cross sections and to measure prompt gamma-ray yields. Such experiments require substantial space due to the numerous detectors and shielding used and demand stable and reproducible beam conditions from day to day.

The two examples illustrate that, with its two beamlines, the OncoRay experimental room allows the entire spectrum of medical physics translational research - from initial laboratory experiments to realistic clinical irradiation scenarios - to be covered.

The facility is also frequently used by external users. Examples of experiments by external users performed in the OncoRay experimental room include a group from the German national metrology institute (Physikalisch-Technische Bundesanstalt, PTB, Braunschweig) who measured spectra of secondary neutrons produced by the proton beam in experiments performed at the FB-BL (59, 60). Researchers from the German Cancer Research Center (DKFZ) in Heidelberg irradiated water samples at the FB-BL to characterize the oxygen depletion rate by proton beams at different dose rates (61). The company ADVACAM from Prague, Czech Republic, has made use of the FB-BL to test their detectors for proton out-of-field dosimetry at conventional and ultra-high dose rates (62). Scientists from Helmholtz-Zentrum Dresden-Rossendorf make regular use of the FB-BL to test and calibrate their instruments used for beam diagnostics in laser-plasma acceleration experiments (63–65). Researchers from Technical University and Helmholtz Center Munich designed a proton minibeam setup and implemented it at the FB-BL for radiobiological experiments (66). In addition to that, radiation hardness of electronic components was studied by developers from academia and industry using dedicated experimental setups at the FB-BL. Furthermore, practical training sessions for future medical physics experts enrolled in OncoRay’s Medical Radiation Sciences Master course are held in the experimental room of the Dresden proton therapy facility.

## 6 Summary and outlook

This article describes the beamlines in the experimental room of the Dresden proton therapy facility, their characteristics and the available infrastructure for experiments.

The facility has unique features, providing two complementary beamlines with different capabilities (a clinical-like beamline with pencil beam scanning option and another one providing static pencil beams with an extended parameter range) installed in a large experimental room that provides enough space for the installation of large-scale devices. The close vicinity to the clinical gantry room creates an ideal environment for future translation of developed methods and devices to application in patients.

Future plans for the facility include the installation of a beam gating interface for the PBS-BL. The experimental capabilities for UHDR experiments at the FB-BL should be further improved by increasing the available maximum beam current and improving the beam monitor systems.

Besides use by OncoRay researchers, the proton beams are also available for external researchers from academia and industry within collaboration projects. The experimental beam data presented in this article will be useful for the planning and preparation of future experiments.

## Data Availability

The raw data supporting the conclusions of this article will be made available by the authors, without undue reservation.

## References

[1] Henthorn NT Sokol O Durante M De Marzi L Pouzoulet F Miszczyk J. Mapping the future of particle radiobiology in europe: The inspire project. Front Phys. (2020) 8:565055. doi: 10.3389/fphy.2020.565055

[2] Aylward JD Henthorn N Manger S Warmenhoven JW Merchant MJ Taylor MJ. Characterisation of the uk high energy proton research beamline for high and ultra-high dose rate (flash) irradiation. Biomed Phys Eng Express. (2023) 9:055032. doi: 10.1088/2057-1976/acef25 37567152

[3] Lis M Newhauser W Donetti M Durante M Weber U Zipfel B. A facility for the research, development, and translation of advanced technologies for ion-beam therapies. J Instrumentation. (2021) 16:T03004. doi: 10.1088/1748-0221/16/03/T03004

[4] Rovituso M Groenendijk C van der Wal E van Burik W Ibrahimi A Rituerto Prieto H. Characterisation of the hollandptc r&d proton beamline for physics and radiobiology studies. Physica Med. (2025) 130:104883. doi: 10.1016/j.ejmp.2024.104883 39778326

[5] Zink K Baumann KS Theiss U Subtil F Lahrmann S Eberle F. Organization and operation of multi particle therapy facilities: the marburg ion-beam therapy center. Health Technol. (2024) 929–38. doi: 10.1007/s12553-024-00881-4 PMC11358185 39219555

[6] Haberer T Debus J Eickhoff H Jäkel O Schulz-Ertner D Weber U. The heidelberg ion therapy center. Radiother Oncol. (2004) 73:S186–90. doi: 10.1016/S0167-8140(04)80046-X 15971340

[7] Tommasino F Rovituso M Fabiano S Piffer S Manea C Lorentini S. Proton beam characterization in the experimental room of the trento proton therapy facility. Nucl Instruments Methods Phys Res Section A: Accelerators Spectrometers Detectors Associated Equip. (2017) 869:15–20. doi: 10.1016/j.nima.2017.06.017

[8] Tommasino F Rovituso M Bortoli E La Tessa C Petringa G Lorentini S. A new facility for proton radiobiology at the trento proton therapy centre: Design and implementation. Physica Med. (2019) 58:99–106. doi: 10.1016/j.ejmp.2019.02.001 30824157

[9] Rossi S. The national centre for oncological hadrontherapy (cnao): Status and perspectives. Physica Med. (2015) 31:333–51. doi: 10.1016/j.ejmp.2015.03.001 25840619

[10] Diffenderfer ES Verginadis II Kim MM Shoniyozov K Velalopoulou A Goia D. Design, implementation, and *in-vivo* validation of a novel proton flash radiation therapy system. Int J Radiat OncologyBiologyPhysics. (2020) 106:440–8. doi: 10.1016/j.ijrobp.2019.10.049 PMC7325740 31928642

[11] Patriarca A Fouillade C Auger M Martin F Pouzoulet F Nauraye C. Experimental set-up for flash proton irradiation of small animals using a clinical system. Int J Radiat OncologyBiologyPhysics. (2018) 102:619–26. doi: 10.1016/j.ijrobp.2018.06.403 30017793

[12] Stock M Georg P Mayer R Böhlen TT Vatnitsky S. Development of clinical programs for carbon ion beam therapy at medaustron. Int J Particle Ther. (2015) 2:474–7. doi: 10.14338/IJPT-15-00022.1 PMC6874201 31772958

[13] Helmbrecht S Baumann M Enghardt W Fiedler F Krause M Lühr A. Design and implementation of a robust and cost-effective double-scattering system at a horizontal proton beamline. J Instrumentation. (2016) 11:T11001. doi: 10.1088/1748-0221/11/11/T11001

[14] Vandeplassche D Beeckman W Zaremba S Jongen Y Tachikawa T. Extracted beams from IBA’s C235. Conf Proc C. (1997) 970512:1045–7. doi: 10.1109/PAC.1997.749923

[15] Petzoldt J Roemer KE Enghardt W Fiedler F Golnik C Hueso-González F. Characterization of the microbunch time structure of proton pencil beams at a clinical treatment facility. Phys Med Biol. (2016) 61:2432. doi: 10.1088/0031-9155/61/6/2432 26943881

[16] Marchand B Prieels D Bauvi B R Sépulchre MG. Iba proton pencil beam scanning: An innovative solution for cancer treatment. Proc EPAC 2000 Vienna Austria. (2000), 2539–41.

[17] Kormoll T Duplicy A Enghardt W Helmbrecht S Hueso Gonzalez F. 106: A beam control system for an experimental beam line operated parallel to a therapeutic beam line. Radiother Oncol. (2014) 110:S52–3. doi: 10.1016/S0167-8140(15)34127-X

[18] Beyreuther E Baumann M Enghardt W Helmbrecht S Karsch L Krause M. Research facility for radiobiological studies at the university proton therapy dresden. Int J Particle Ther. (2018) 5:172–82. doi: 10.14338/IJPT-18-00008.1 PMC6871594 31773028

[19] Horst F Beyreuther E Bodenstein E Gantz S Misseroni D Pugno NM. Passive sobp generation from a static proton pencil beam using 3d-printed range modulators for flash experiments. Front Phys. (2023) 11:1213779. doi: 10.3389/fphy.2023.1213779

[20] Helmbrecht S Fiedler F Meyer M Kaever P Kormoll T. 124 - proton beams for physics experiments at oncoray. Radiother Oncol. (2016) 118:S60–1. doi: 10.1016/S0167-8140(16)30124-4

[21] Metzkes-Ng J Brack FE Kroll F Bernert C Bock S Bodenstein E. The Dresden platform is a research hub for ultra-high dose rate radiobiology. Sci Rep. (2023) 13:20611. doi: 10.1038/s41598-023-46873-8 37996453 PMC10667545

[22] Suckert T Müller J Beyreuther E Azadegan B Brüggemann A Bütof R. High-precision image-guided proton irradiation of mouse brain sub-volumes. Radiother Oncol. (2020) 146:205–12. doi: 10.1016/j.radonc.2020.02.023 32222488

[23] Bäumer C Koska B Lambert J Timmermann B Mertens T Talla PT. Evaluation of detectors for acquisition of pristine depth-dose curves in pencil beam scanning. J Appl Clin Med Phys. (2015) 16:151–63. doi: 10.1120/jacmp.v16i6.5577 PMC5691023 26699567

[24] Farr JB Dessy F De Wilde O Bietzer O Schönenberg D. Fundamental radiological and geometric performance of two types of proton beam modulated discrete scanning systems. Med Phys. (2013) 40:072101. doi: 10.1118/1.4807643 23822445

[25] Courtois C Boissonnat G Brusasco C Colin J Cussol D Fontbonne J. Characterization and performances of a monitoring ionization chamber dedicated to iba-universal irradiation head for pencil beam scanning. Nucl Instruments Methods Phys Res Section A: Accelerators Spectrometers Detectors Associated Equip. (2014) 736:112–7. doi: 10.1016/j.nima.2013.10.014

[26] Janson M Glimelius L Fredriksson A Traneus E Engwall E. Treatment planning of scanned proton beams in raystation. Med Dosimetry. (2024) 49:2–12. doi: 10.1016/j.meddos.2023.10.009 37996354

[27] Oborn BM Semioshkina E van der Kraaij E Hoffmann AL. Simulation and experimental benchmarking of a proton pencil beam scanning nozzle model for development of mr-integrated proton therapy. Med Phys. (2024) 6196–205. doi: 10.1002/mp.17279 38949569

[28] Pape K Lößner AJ William D Czempiel T Beyreuther E Klimova A. Sensitization of patient derived colorectal cancer organoids to photon and proton radiation by targeting dna damage response mechanisms. Cancers. (2022) 14(20):4984. doi: 10.3390/cancers14204984 36291768 PMC9599341

[29] Suckert T Rassamegevanon T Müller J Dietrich A Graja A Reiche M. Applying tissue slice culture in cancer research—insights from preclinical proton radiotherapy. Cancers. (2020) 12(6):1589. doi: 10.3390/cancers12061589 32560230 PMC7352770

[30] Tillner F Thute P Löck S Dietrich A Fursov A Haase R. Precise image-guided irradiation of small animals: a flexible non-profit platform. Phys Med Biol. (2016) 61:3084. doi: 10.1088/0031-9155/61/8/3084 27008208

[31] Müller J Schürer M Neubert C Tillner F Beyreuther E Suckert T. Multi-modality bedding platform for combined imaging and irradiation of mice. Biomed Phys Eng Express. (2020) 6:037003. doi: 10.1088/2057-1976/ab79f1 33438682

[32] Schneider M Bodenstein E Bock J Dietrich A Gantz S Heuchel L. Combined proton radiography and irradiation for high-precision preclinical studies in small animals. Front Oncol. (2022) 12:982417. doi: 10.3389/fonc.2022.982417 36419890 PMC9677333

[33] Schneider M Schilz JD Schürer M Gantz S Dreyer A Rothe G. Sapphire - establishment of small animal proton and photon image-guided radiation experiments. Phys Med Biol. (2024) 69:095020. doi: 10.1088/1361-6560/ad3887 38537301

[34] Görte J Beyreuther E Danen EHJ Cordes N. Comparative proton and photon irradiation combined with pharmacological inhibitors in 3d pancreatic cancer cultures. Cancers. (2020) 12(11):3216. doi: 10.3390/cancers12113216 33142778 PMC7692858

[35] Schniewind I Hadiwikarta WW Grajek J Poleszczuk J Richter S Peitzsch M. Cellular plasticity upon proton irradiation determines tumor cell radiosensitivity. Cell Rep. (2022) 38:110422. doi: 10.1016/j.celrep.2022.110422 35196495

[36] Suckert T Beyreuther E Müller J Azadegan B Meinhardt M Raschke F. Late side effects in normal mouse brain tissue after proton irradiation. Front Oncol. (2021) 10:598360. doi: 10.3389/fonc.2020.598360 33520710 PMC7842140

[37] Szabó ER Brand M Hans S Hideghéty K Karsch L Lessmann E. Radiobiological effects and proton rbe determined by wildtype zebrafish embryos. PloS One. (2018) 13:1–18. doi: 10.1371/journal.pone.0206879 PMC6224071 30408095

[38] Brüchner K Beyreuther E Baumann M Krause M Oppelt M Pawelke J. Tumour irradiation in mice with a laser-accelerated proton beam. Nat Phys. (2022) 18:316–22. doi: 10.1038/s41567-022-01520-3

[39] Beyreuther E Brand M Hans S Hideghéty K Karsch L Leßmann E. Feasibility of proton flash effect tested by zebrafish embryo irradiation. Radiother Oncol. (2019) 139:46–50. doi: 10.1016/j.radonc.2019.06.024 31266652

[40] Karsch L Pawelke J Brand M Hans S Hideghéty K Jansen J. Beam pulse structure and dose rate as determinants for the flash effect observed in zebrafish embryo. Radiother Oncol. (2022) 173:49–54. doi: 10.1016/j.radonc.2022.05.025 35661675

[41] Horst F Bodenstein E Brand M Hans S Karsch L Lessmann E. Dose and dose rate dependence of the tissue sparing effect at ultra-high dose rate studied for proton and electron beams using the zebrafish embryo model. Radiother Oncol. (2024) 194:110197. doi: 10.1016/j.radonc.2024.110197 38447870

[42] Hoffmann A Oborn B Moteabbed M Yan S Bortfeld T Knopf A. MR-guided proton therapy: a review and a preview. Radiat Oncol. (2020) 15:1–13. doi: 10.1186/s13014-020-01571-x PMC7260752 32471500

[43] Schellhammer SM Gantz S Lühr A Oborn BM Bussmann M Hoffmann AL. Technical note: Experimental verification of magnetic field-induced beam deflection and bragg peak displacement for mr-integrated proton therapy. Med Phys. (2018) 45:3429–34. doi: 10.1002/mp.12961 29763970

[44] Lühr A Burigo LN Gantz S Schellhammer SM Hoffmann AL. Proton beam electron return effect: Monte carlo simulations and experimental verification. Phys Med Biol. (2019) 64:035012. doi: 10.1088/1361-6560/aafab4 30577039

[45] Causer TJ Schellhammer SM Gantz S Lühr A Hoffmann AL Metcalfe PE. First application of a high-resolution silicon detector for proton beam bragg peak detection in a 0. 95 t magnetic field Med Phys. (2020) 47:181–9. doi: 10.1002/mp.13871 31621914

[46] Schellhammer SM Hoffmann AL Gantz S Smeets J van der Kraaij E Quets S. Integrating a low-field open mr scanner with a static proton research beam line: proof of concept. Phys Med Biol. (2018) 63:23LT01. doi: 10.1088/1361-6560/aaece8 30465549

[47] Gantz S Karsch L Pawelke J Schieferecke J Schellhammer S Smeets J. Direct visualization of proton beam irradiation effects in liquids by mri. Proc Natl Acad Sci. (2023) 120:e2301160120. doi: 10.1073/pnas.2301160120 37252953 PMC10265969

[48] Gantz S Hietschold V Hoffmann AL. Characterization of magnetic interference and image artefacts during simultaneous in-beam mr imaging and proton pencil beam scanning. Phys Med Biol. (2020) 65:215014. doi: 10.1088/1361-6560/abb16f 33151908

[49] Gebauer B Pawelke J Hoffmann A Lühr A. Technical note: Experimental dosimetric characterization of proton pencil beam distortion in a perpendicular magnetic field of an in-beam mr scanner. Med Phys. (2023) 50:7294–303. doi: 10.1002/mp.16448 37161832

[50] Joint press release by the dresden university hospital and hzdr on January 9th, 2024 (2024). Available online at: https://www.hzdr.de/db/Cms?pOid=71010&pNid=3438 (Accessed December 4, 2024).

[51] Pausch G Berthold J Enghardt W Römer K Straessner A Wagner A. Detection systems for range monitoring in proton therapy: Needs and challenges. Nucl Instr Meth A. (2018) 161227. doi: 10.1016/j.nima.2018.09.062

[52] Golnik C Hueso-González F Müller A Dendooven P Enghardt W Fiedler F. Range assessment in particle therapy based on prompt *γ*-ray timing measurements. Phys Med Biol. (2014) 59:5399. doi: 10.1088/0031-9155/59/18/5399 25157685

[53] Werner T Berthold J Enghardt W Hueso-Gonzalez F Kogler T Petzoldt J. (2017). Range verification in proton therapy by prompt gamma-ray timing (pgt): Steps towards clinical implementation, in: IEEE Nuclear Science Symposium and Medical Imaging Conference (NSS/MIC), . pp. 1–5. IEEE. doi: 10.1109/nssmic.2017.8532807

[54] Werner T Berthold J Hueso-González F Kögler T Petzoldt J Roemer K. Processing of prompt gamma-ray timing data for proton range measurements at a clinical beam delivery. Phys Med Biol. (2019) 64:105023. doi: 10.1088/1361-6560/ab176d 30965311

[55] Pausch G Müller C Berthold J Enghardt W Küchler M Römer KE. (2018). Effect of strong load variations on gain and timing of cebr3 scintillation detectors used for range monitoring in proton radiotherapy, in: IEEE Nuclear Science Symposium and Medical Imaging Conference (NSS/MIC), Sydney, Australia.

[56] Roemer KE Pausch G Aldawood S Berthel M Dreyer A Enghardt W. (2014). Scintillator characterization at energies relevant for a prompt gamma detection system in particle therapy, in: IEEE Nuclear Science Symposium and Medical Imaging Conference (NSS/MIC), . pp. 1–2. IEEE. doi: 10.1109/nssmic.2014.7431196

[57] Permatasari FF. Development of a prompt γ-ray timing system including a proton bunch monitor for range verification in proton therapy. Dresden, Germany: TUD Dresden University of Technology (2023).

[58] Makarevich K Schellhammer SM Pausch G Römer KE Tiebel J Turko J. Proton bunch monitors for the clinical translation of prompt gamma-ray timing. Phys Med Biol. (2024) 69:225013. doi: 10.1088/1361-6560/ad8c96 39530455

[59] Dommert M Reginatto M Zboril M Fiedler F Helmbrecht S Enghardt W. Measurement of the energy spectrum of secondary neutrons in a proton therapy environment. Curr Dir Biomed Eng. (2017) 3:83–6. doi: 10.1515/cdbme-2017-0018

[60] Dommert M Reginatto M Zbořil M Lutz B. Dead time corrections for bonner sphere measurements of secondary neutrons at a proton therapy facility. J Instrumentation. (2021) 16:P03038. doi: 10.1088/1748-0221/16/03/P03038

[61] Jansen J Knoll J Beyreuther E Pawelke J Skuza R Hanley R. Does flash deplete oxygen? experimental evaluation for photons, protons, and carbon ions. Med Phys. (2021) 48:3982–90. doi: 10.1002/mp.14917 33948958

[62] Oancea C Granja C Marek L Jakubek J Šolc J Bodenstein E. Out-of-field measurements and simulations of a proton pencil beam in a wide range of dose rates using a timepix3 detector: Dose rate, flux and let. Physica Med. (2023) 106:102529. doi: 10.1016/j.ejmp.2023.102529 36657235

[63] Reimold M Assenbaum S Beyreuther E Bodenstein E Brack FE Eisenmann C. Octopod: single-bunch tomography for angular-spectral characterization of laser-driven protons. High Power Laser Sci Eng. (2023) 11:e68. doi: 10.1017/hpl.2023.55

[64] Corvino A Reimold M Beyreuther E Brack FE Kroll F Pawelke J. miniscidom: a scintillatorbased tomograph for volumetric dose reconstruction of single laser-driven proton bunches. High Power Laser Sci Eng. (2024) 12:e17. doi: 10.1017/hpl.2024.1

[65] Schilz JD Bodenstein E Brack FE Horst F Irman A Kroll F. Absolute energy-dependent scintillating screen calibration for real-time detection of laser-accelerated proton bunches. Rev Sci Instruments. (2024) 95:073303. doi: 10.1063/5.0206931 39058268

[66] Ahmed M Beyreuther E Gantz S Horst F Meyer J Pawelke J. Design and dosimetric characterization of a transportable proton minibeam collimation system. Front Oncol. (2024) 14:1473625. doi: 10.3389/fonc.2024.1473625 39741979 PMC11685229

